# Supplementary material for: SETDB1 prevents TET2-dependent activation of IAP retroelements in naïve embryonic stem cells
Source: Genome Biol. 2018 Jan 19;19:6. doi: 10.1186/s13059-017-1376-y (PMC5775534; doi:10.1186/s13059-017-1376-y)
Supplement: Supplementary file 1 — Figure S1. SETDB1 regulates ERV silencing in naïve ESCs. Figure S2. Removal of TET2 dampens SETDB1-mediated IAP activation in naïve cells. Figure S3. SETDB1-mediated IAP activation is not linked to DNA methylation changes. Figure S4. SETDB1 depletion does not lead to DNA methylation changes. Figure S5. TET2 activity is associated with loss of H4R3me2s at IAPs. (PDF 828 kb) [file 13059_2017_1376_MOESM1_ESM.pdf]

Supplementary Figures

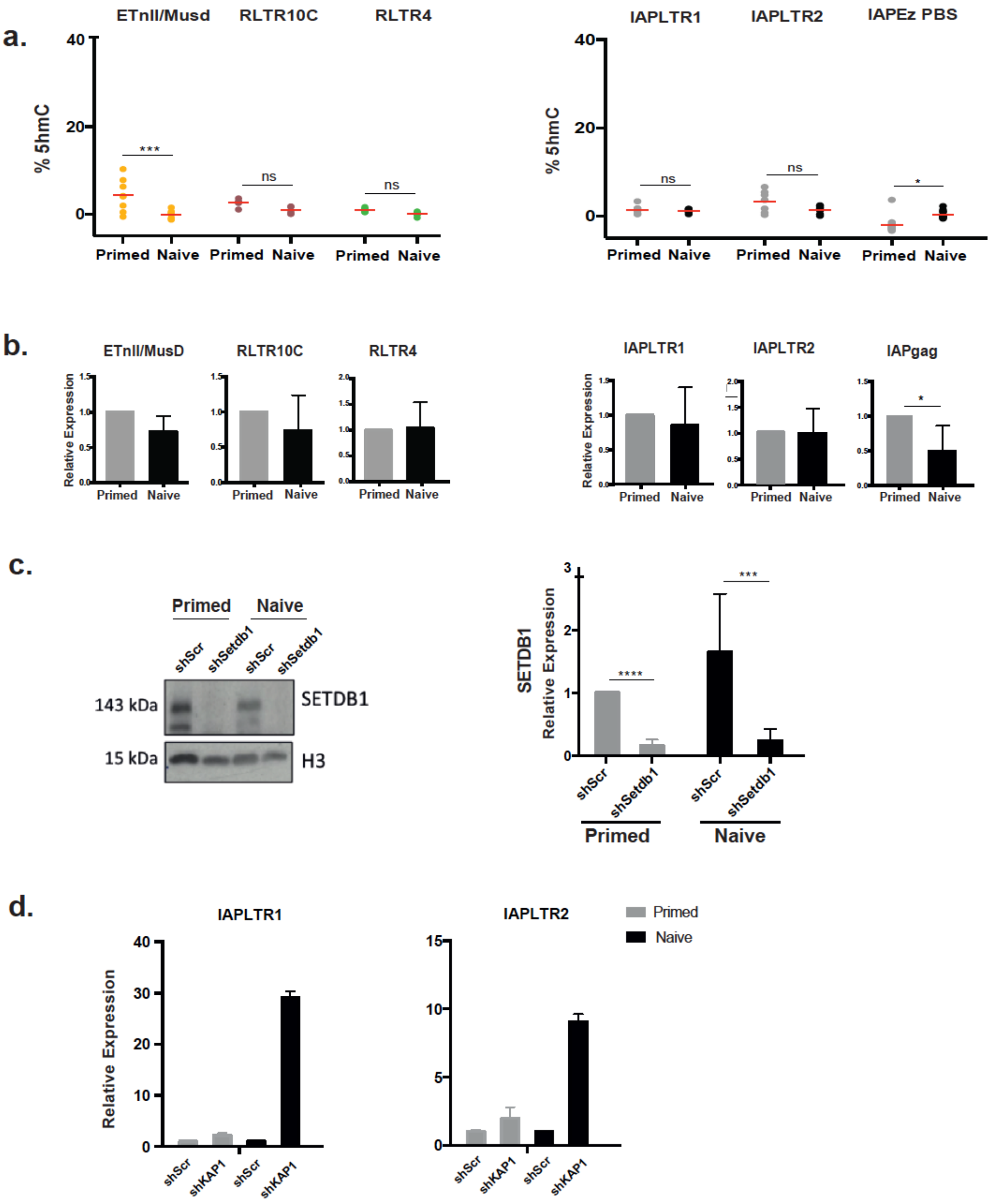

Supplementary Figure 1 – SETDB1 regulates ERV silencing in naive ESCs.
a. Deep amplicon sequencing from oxBS-treated DNA was used to measure 5hmC levels at ERVs in primed and naive ESCs; each data point represents the average value from two biological replicates at a given CpG within the amplicon (ANOVA with Tukey's multiple comparison test, * p<0.05, *** p<0.001, **** p<0.0001). **b.** RT-qPCR data of primed and naïve ESCs. Each bar represents mean values and error bars indicate s.d. (n=3-10; t-test, * p<0.05). **c.** Western blot (representative data from n=2) and RT-qPCR analyses (n=7) show SETDB1 depletion by lentiviral delivery of shRNAs (ANOVA with Tukey's multiple comparison test, *** p<0.001, **** p<0.0001). **d.** RT-qPCR data of KAP1 depleted ESCs at IAPs in primed and naïve conditions.

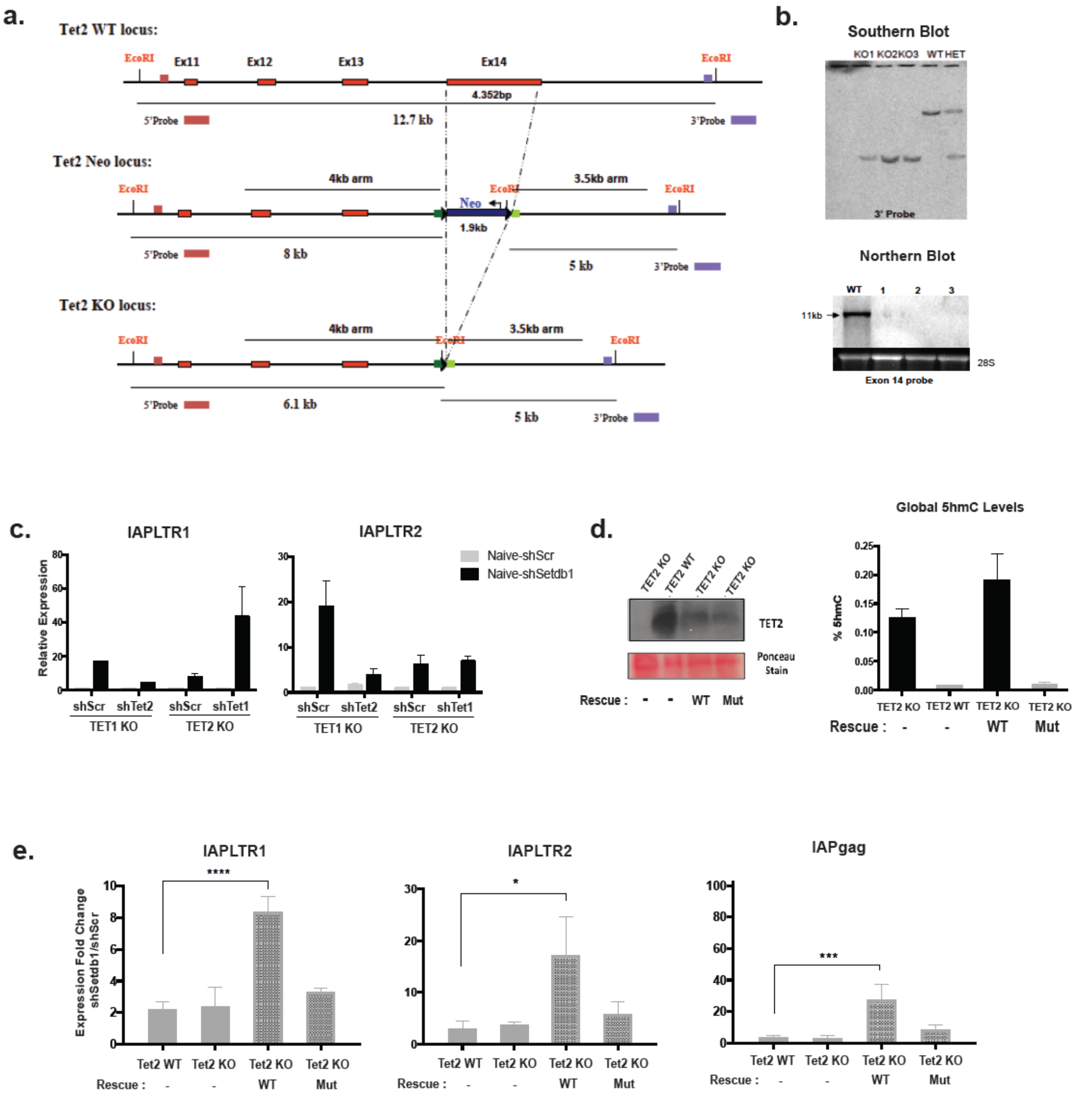

Supplementary Figure 2 – Removal of TET2 dampens SETDB1-mediated IAP activation in naïve cells. a. *Tet2* KO ESC targeting strategy. A 12.7kb genomic region containing *Tet2* exons 11 to 14 is depicted. *Tet2* exon 14 was substituted by a loxP-PGK-Neo-p(A)-loxP cassette by homologous recombination and then deleted by Cre-mediated recombination. EcoRI digestion fragments were used for Southern blot analysis using the 5' and 3' probes depicted. **b.** Southern blot analysis using the 3' probe shows correct targeting of *Tet2* locus. Northern blot analysis using a probe for exon 14 is unable to detect RNA in three independent *Tet2* KO ES clones. **c.** Relative expression of IAPs in *Tet2*-depleted *Tet1* KO ESCs or, reciprocally, in *Tet1*-depleted *Tet2* KO ESC, in 2i conditions. The error bars indicate s.d. from three technical replicates. **d.** Western blot reveals similar level of TET2 expression in the *Tet2* rescue lines (representative replicate of n=2). Global 5hmC levels reveals similar levels between WT *Tet2* ESCs and cells rescued with WT TET2, whereas TET2 KO ESCs and cells rescued with the mutant TET2 has low levels of 5hmC. **e.** RT-qPCR analysis of *Tet2* WT, KO and rescue cell lines at IAPs in primed ESCs (n=3-4; ANOVA with Tukey's multiple comparison test, * $p<0.05$, *** $p<0.001$, **** $p<0.0001$). The results are presented as the ratio of shSETDB1/shScr and mean ± s.d.

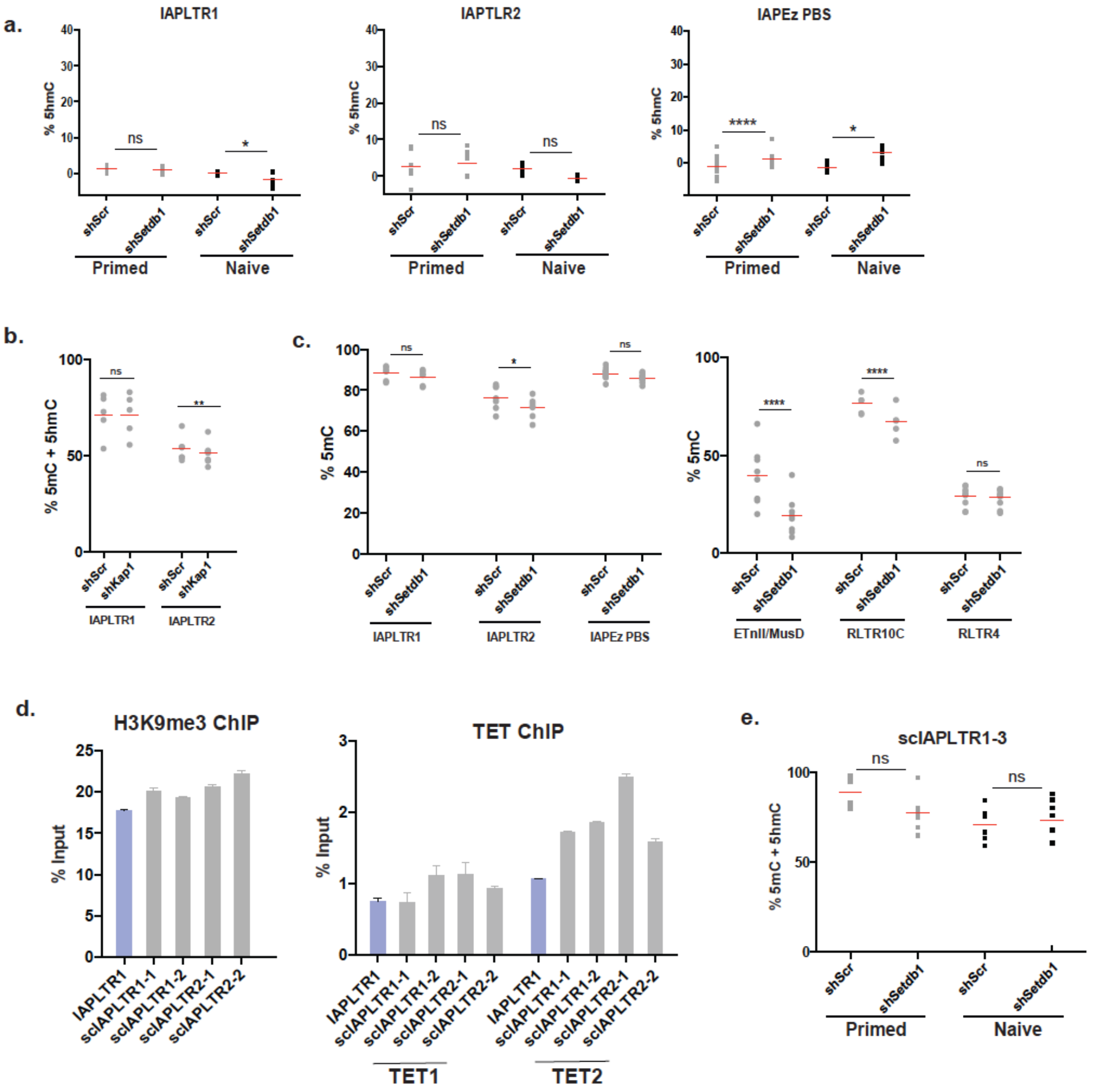

Supplementary Figure 3 – SETDB1-mediated IAP activation is not linked to DNA methylation changes. a-c. 5hmC levels in primed and naïve conditions upon SETDB1 knockdown (a), 5mC+5hmC levels in naïve ESCs upon KAP1 knockdown (b), 5mC levels in primed ESCs upon SETDB1 knockdown (c) at IAPs; each data point represents the average value from two biological replicates at a given CpG within the amplicon (ANOVA with Tukey's multiple comparison test, * p<0.05, ** p<0.01, **** p<0.0001). **d.** H3K9me3, TET1 and TET2 enrichment across individual IAP copies in primed ESCs compared to pool of IAP copies (blue bars). **e.** 5mC+5hmC levels upon SETDB1 removal at individual IAPs in primed and naïve conditions (n=2).

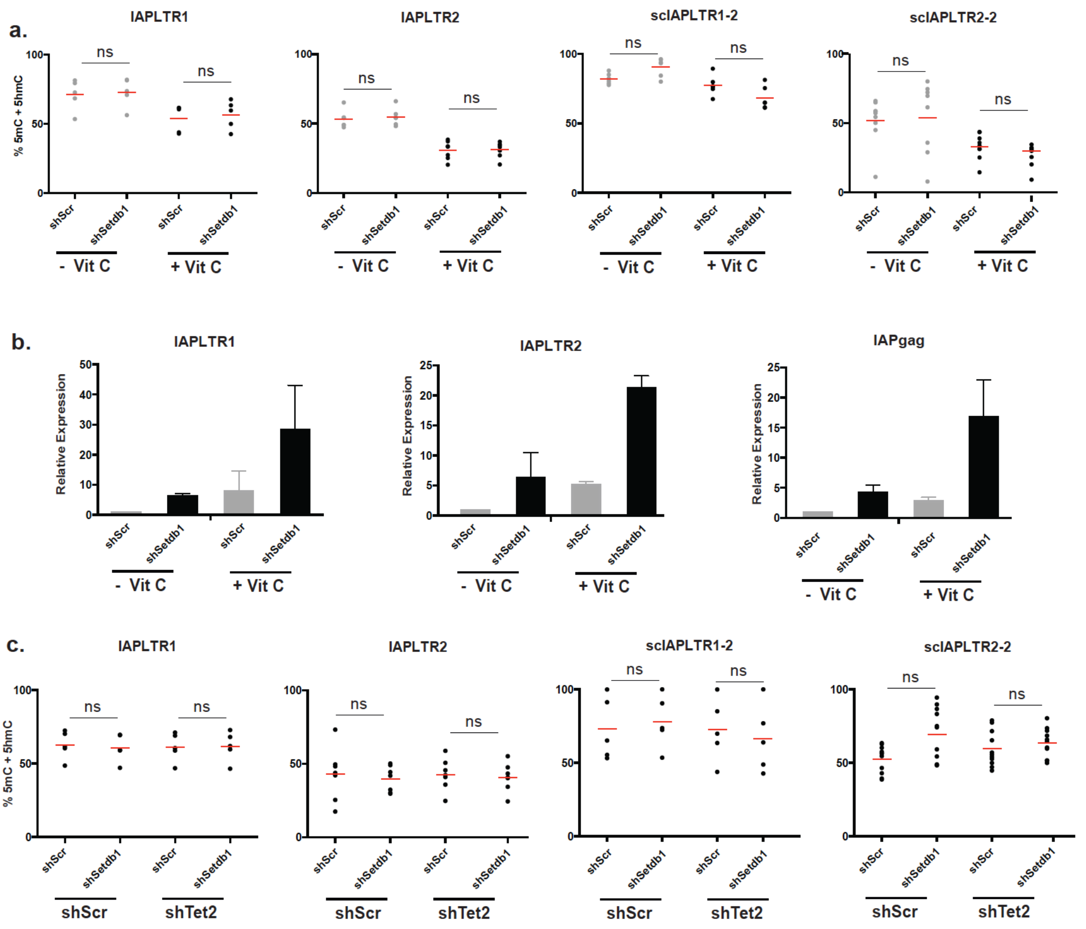

Supplementary Figure 4 – SETDB1-depletion does not lead to DNA methylation changes. a. 5mC+5hmC levels in naïve ESCs upon SETDB1 knockdown with and without vitamin C at IAPs; each data point represents the average value from two biological replicates at a given CpG within the amplicon (ANOVA with Tukey's multiple comparison test, * p<0.05, ** p<0.01, **** p<0.0001).**b.** RT-qPCR data of SETDB1 depleted ESCs at IAPs in naïve conditions with and without vitamin C. **c.** 5mC+5hmC levels upon SETDB1 removal at IAP pools and individual IAP copies in *Tet2* KD naïve cells (n=1); each data point represents the value at a given CpG within the amplicon (ANOVA with Tukey's multiple comparison test).

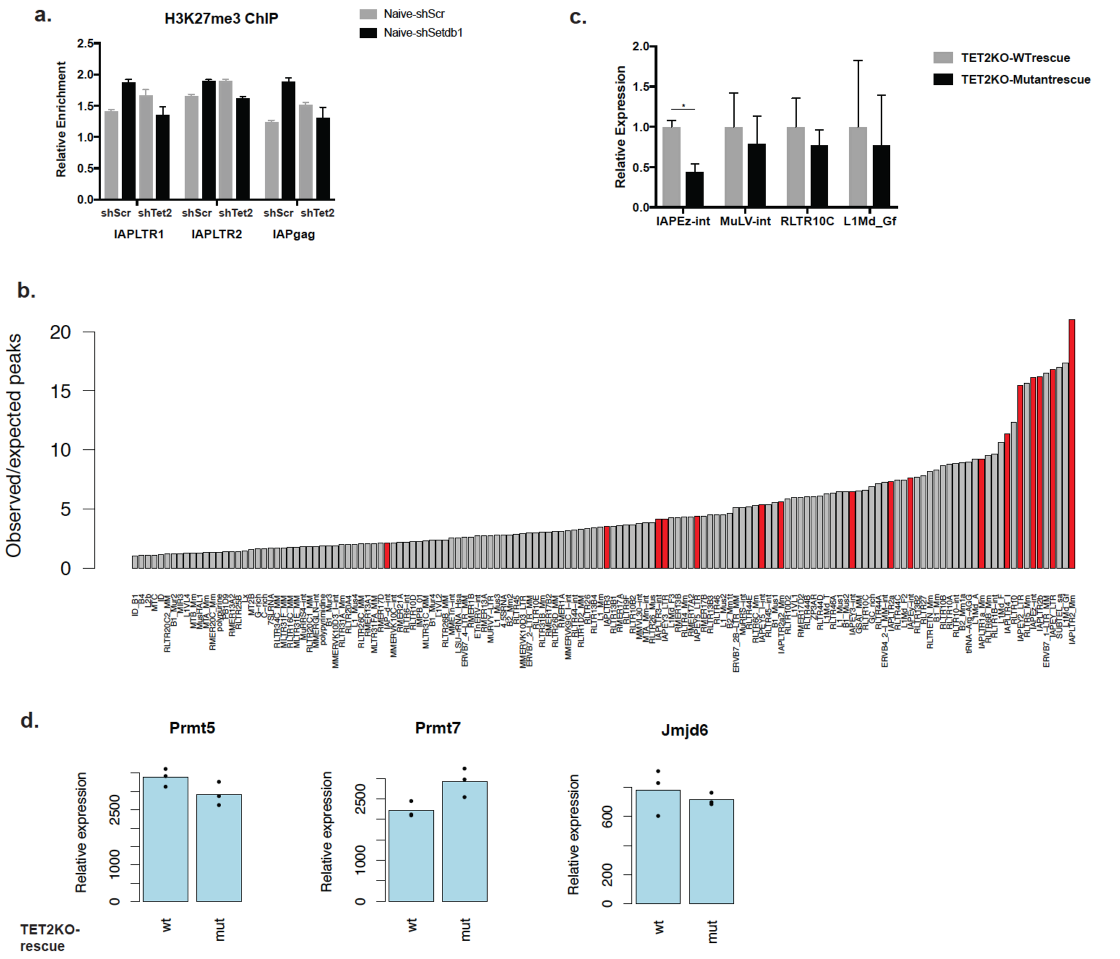

Supplementary Figure 5 – TET2 activity is associated with loss of H4R3me2 at IAPs. a. ChIP-qPCR data for H3K27me3 at IAPs upon SETDB1 loss in the presence and absence of TET2 (n=2-3). **b.** Repeat classes that are enriched for H4R3me2s in naïve ESCs. IAP elements are indicated in red **c.** Relative expression of transposons in Tet2 rescue cell lines based on RNA-seq data (n=3, * p<0.05) **d.** RNA-seq data of Prmt5, Prmt7 and Jmjd6 genes in Tet2 rescue lines upon SETDB1 depletion.
